# A Weekly, Evidence-Based Health Letter for Caregivers (90Second Caregiver): Usability Study

**DOI:** 10.2196/14496

**Published:** 2020-02-12

**Authors:** Athena Milios, Patrick McGrath, Hannah Baillie

**Affiliations:** 1 Department of Psychiatry, Dalhousie University Halifax, NS Canada; 2 Centre for Research in Family Health, IWK Health Centre Halifax, NS Canada

**Keywords:** caregivers, mental health, usability, depression, anxiety, stigma, hope, health information, persuasive design

## Abstract

**Background:**

Informal caregivers are family members or close friends who provide unpaid help to individuals with acute or chronic health conditions so that they can manage daily life tasks. The greatest source of health information is the internet for meeting the needs of caregivers. However, information on the internet may not be scientifically valid, it may be written in language that is difficult to read, and is often in very large doses. 90Second Caregiver is a health letter whose aim is to disseminate knowledge to caregivers in a user-friendly, weekly format, in order to improve their wellbeing.

**Objective:**

The main objective was to test a sample of 90Second Caregiver health letters in order to assess their usability and to optimize the design and content of the health letters.

**Methods:**

Usability research themes were assessed using semi-structured phone interviews, incorporating the Think Aloud method with retrospective questioning.

**Results:**

Usability was assessed in the context of five main themes: understandability and learnability, completeness, relevance, and quality and credibility of the health letter content, as well as design and format. Caregivers generally provided positive feedback regarding the usability of the letters. The usability feedback was used to refine 90Second Caregiver in order to improve the design and content of the series. Based on the results of this study, it may be of maximum benefit to target the series towards individuals who are new to caregiving or part-time caregivers, given that these caregivers of the sample found the letters more useful and relevant and had the most positive usability experiences.

**Conclusions:**

The findings assisted in the improvement of the 90Second Caregiver template, which will be used to create future health letters and refine the letters that have already been created. The findings have implications for who the 90Second Caregiver series should be targeting (ie, newer or part-time caregivers) in order to be maximally impactful in improving mental health and wellbeing-related outcomes for caregivers, such as self-efficacy and caregiving knowledge. The results of this study may be generalizable to the examination of other electronic health information formats, making them valuable to future researchers testing the usability of health information products. In addition, the methods used in this study are useful for usability hypothesis generation. Lastly, our 90Second delivery approach can generate information useful for a set of similar products (eg, weekly health letters targeted towards other conditions/populations).

## Introduction

### Background and Rationale

Informal caregivers are family members or close friends who provide unpaid help to individuals with acute or chronic health conditions so that they can manage daily life tasks [1]. Although informal caregiving is better than paid caregiving for the mental and physical well-being of the individuals receiving the care, it can negatively affect the well-being of the caregivers themselves [1]. Caregivers have been found to have high levels of stress, depression, and risk for mortality. They are less likely to preventatively manage their own health, that is, to take care of themselves through engaging in healthy lifestyle behaviors such as exercise, healthy diet, and proper sleep hygiene [1,2]. A meta-analysis found that caregivers, compared with noncaregivers, had higher stress levels and depressive symptoms, lower self-efficacy, and poorer general subjective well-being [2,3]. Self-efficacy is the belief that one is capable and competent to manage situations. Self-efficacy in caregivers is associated with a lower risk of caregiver burnout and psychological distress and higher care-recipient well-being [4].

The greatest source of health information for caregivers is the internet. However, there are many barriers facing caregivers given the amount of scientific knowledge available to the public and the difficulty in interpreting dense (low readability) and lengthy (containing information in large doses) scientific articles [5,6]. Furthermore, much of the information on the internet is not evidence based or scientifically valid [5,6]. Caregivers of those with mental and physical health problems are very busy and stressed and need a trustworthy source of easy-to-read, concise, accurate, and evidence-based information presented in manageable portions. Exposure to inaccurate, misleading, outdated, or vague information could be detrimental to caregiver and patient health-related outcomes [6].

90Second Caregiver is a health letter that aims to disseminate knowledge to caregivers in a user-friendly, weekly format to improve their self-efficacy, increase their health-related caregiving knowledge (on a general level, with the goal of them being able to apply this general knowledge to disease-specific caregiving dilemmas), and promote healthy coping behaviors. The letters are all developed using credible scientific sources, such as academic journals and/or government agencies.

Usability research involves the participants using and evaluating a product or service, such as Web-based electronic health (eHealth) apps, websites, or health documents. Usability studies aim to detect usage-related difficulties and improve the design of health-related services and products [7].

### Objectives

The main objective was to pilot test a sample of 90Second Caregiver health letters to obtain data regarding their usability and to optimize the delivery, design, and content utility of the health letters. Usability was assessed in terms of design and format, understandability and learnability, completeness, practical relevance, and quality and credibility of the health letter content. The secondary objectives were to assess factors that participants liked and disliked about the format and content, to determine which additional topics participants would like to see in the future, to assess their interest in becoming subscribers in the future, and to determine what improvements and changes the caregivers would like to see regarding the design and format or content of the health letters.

## Methods

### Recruitment

After the research ethics board’s approval was obtained, participants eligible for the study were recruited through our health center’s volunteer service and the Brain Injury Association of Nova Scotia. A sample size calculation was not performed because most usability problems are discovered by the first 5 participants [8].

The exclusion criteria were as follows: (1) individuals who were not primary caregivers, (2) participants who did not speak English, (3) participants who did not have access to a computer or email or internet, and (4) participants who did not have a phone. The 90Second health letters were delivered by an email link to a Web page.

The first page of the letters had 3 components: 200 words of evidence-based, plain-language health information on a focused issue related to caregiving (the main body), 100 to 150 words of actionable suggestions, and a license-free graphic relating to the topic that supports the main message of each letter (located above the main body). The second page contained a 7-item assessment tool. Each item was rated on a scale from 1 to 5 to generate a total score. There was also an explanation of the total score. The third page was a personal account of a caregiver on the issue (up to 300 words). A 3-item rating of the health letter followed. Finally, links to additional resources for caregivers were provided.

The questions in the 7-item assessment tool capture a single construct (either behavior or attitudes, not simply factual knowledge) based on the central concept of each health letter. The 7 questions can be answered on a scale from 1 (false) to 5 (true) to generate a total score ranging from 7 to 35. Some items can be reverse scored. Items must measure a single construct, should be short (less than 10 words), and not have long or unusual words. Double negatives are not permitted. For example, “I am proud of my role and abilities as a caregiver” is an item in the stigma health letter assessment tool.

The *Rate our Health Letter* scale contains 3 questions/items: “Did you find this health letter helpful?,” “Could you relate to the content of this health letter?,” and “Would you recommend this letter to a friend or organization?” The response options are yes, not applicable, and no. Then, the reader is asked if they have suggestions for future topics.

### Participant Characteristics

The sample included 10 caregivers who evaluated 2 health letters each. The first 5 caregivers evaluated one set of (2) health letters (anxiety and depression), and the second 5 caregivers evaluated another set of (2) health letters (hope and caregiver stigma).

The caregivers’ demographic information is shown in Table 1. The population was a diverse sample of caregivers, caring for individuals with various mental and physical health conditions.

**Table 1:** Demographic characteristics of participants.

Participant	Age (years)	Sex	Length of time spent caregiving	Occupation	Highest level of education	Hours spent caregiving per week	Nature of loved one’s health condition
SL	25	Female	6 months	Nurse	BSc^a^	3	Cancer
SK	52	Male	2 years	Professor	PhD^b^	20	Cancer (breast)
AB	24	Male	1 year	Student	BSc	3	Irritable bowel syndrome
RM	37	Female	3 years	Economist	MA^c^	60	Cancer (leukemia)
ME	43	Female	10 years	Community relations and television producer	MA	3	Epilepsy
KL	34	Female	3 years	Senior policy analyst	Community college diploma	3	Acquired brain injury (traumatic)
JM	57	Female	30 years	Accountant	Postgraduate diploma	25	15q duplication syndrome (neurodevelopmental disorder)
DM	53	Female	4 years	Nurse	Community college (nursing) diploma	60	Acquired brain injury
WM	61	Female	6 years	Administrative assistant	High school	80	Acquired brain injury (anoxic)
CM	77	Female	26 years	Research scientist	PhD	80	Acquired brain injury (traumatic)

^a^BSc: Bachelor of Science.

^b^PhD: Doctor of Philosophy.

^c^MA: Master of Arts.

### Procedure

First, caregivers who had expressed an interest were sent an email with some background information regarding the study and what was involved. The information and consent form was attached to this email. Participants were asked to respond with the dates and times that they would be available for the phone interview.

At the beginning of the phone interview, informed consent was obtained. Participants were then asked questions, regarding (1) age and sex, (2) length of time as a caregiver, (3) current occupation, (4) level of education completed, (5) the nature of their care recipient’s health condition, and (6) length of time spent caregiving per week (Table 1).

Participants were emailed PDF copies of the two 90Second Caregiver health letters that they were to assess and encouraged to vocalize their thought processes as they read them, which is known as the concurrent think aloud technique. The think aloud portion of the interview involved the interviewer recording the report of the thinking process as each participant reflected on the content and format of the sample of health letters. The participants were encouraged to express their understanding, beliefs, attitudes, and expectations regarding the sample of health letters that they were reading. The think aloud method has been shown to be the most effective in detecting usability problems, which is why it was chosen for this study [9].

After they read each health letter, the participants were asked a series of questions to assess its design, format, and content to expand and complement the think aloud results (Textbox 1) [10]. The caregivers’ opinions regarding various aspects of the design and content, including ease of use, ease of learning, completeness, practical relevance, usefulness, quality, and credibility were elicited [11,12].

**Semistructured interview questions. (Caregivers were asked questions 1-9 twice, once for each health letter they read. Then, they were asked questions 10-15 after both the letters were read.)**

1. What is your impression regarding the purpose of the health letter?
2. What is the first section you would read to get started?
3. How do you feel about the way the information was presented/formatted? (Is the text big enough, do you like the pictures, the order of the material, etc) [13].
4. How do you feel about the way the information is written? (Writing level, understanding, readability, and unfamiliar terms) [14].
5. Could we do anything to make this topic easier or more enjoyable to read (illustrations, more explanations, short video clips, etc.)?
6. Was the amount of information included enough/not enough, in other words, how complete did you feel each health letter was in covering the topic?
  - Was there anything you found yourself wanting to know that was not included?
  - Was there any part of the health letter that you thought was unnecessary or should be removed?
  - How did you find the length of each health letter? Would you prefer it longer or shorter?
7. How useful and relevant did you find the information?
8. What knowledge have you learned/gained? What did you already know?
9. What did you like/dislike about the content of each health letter [15]?
  - The main body of text?
  - The tips/suggestions?
  - The self-assessment?
  - The personal account?
10. How satisfied are you with the overall quality and reliability of what you read?
11. What was the best part of the health letters? The worst?
12. If you could change anything (about either the design/format or content), what would it be?
13. What additional topics would you like to see if you were a subscriber to the series?
14. Do you have any suggestions for specific companies, associations, or agencies that you would like to see sponsoring the 90Second Caregiver Series?
15. Would you be interested in subscribing to this series in the future?

The questions were developed by performing a literature search regarding the most important aspects of usability [7]. For example, Lund (2001) found that ease of use, understandability, learnability, usefulness, and overall satisfaction are the most important elements of usability, which is why these themes were incorporated into the question design for this study [11]. The larger list of questions was then narrowed down to a more concise set of questions to eliminate redundancies and ambiguities and reduce the number of questions. Face and content validities of the interview questions were reviewed by a set of psychology researchers to ensure the various domains of usability were adequately covered and to ensure the questions were clear and practical [16].

The think aloud technique was also used to assess how participants answered the assessment questions in each health letter. Caregivers provided feedback on whether the questions in the assessment captured the concept that they were supposed to measure. Hope, caregiver stigma, depression, and anxiety were the topics of the 4 health letters used.

After they completed the phone interview, the participants were sent a thank-you email, with their signed information and consent form and their Can $10 Amazon gift card.

### Data Analysis

The data were deidentified (names and contact information removed) and transcribed. Thematic analysis was performed to analyze the transcripts: frequencies of emerging usability categories and themes from the concurrent think aloud data and the retrospective semistructured usability questions were analyzed to assess the outcome measures of the study (ie, caregiver satisfaction and opinions regarding the main themes of usability—understandability and learnability, completeness, relevance, usefulness, and quality and credibility of the health letter content—as well as design and format) [10].

Usability problems and improvements suggested by participants were also recorded, and their frequencies were analyzed. Modifications were made to 90Second Caregiver based on the participant feedback (see Tables 2 and 3). Combining think aloud data with retrospective questionnaire data is the most complete way to understand the usability experiences of participants [17-19].

**Table 2:** Summary of the usability themes of the Anxiety and Depression health letters’ content and feedback and changes made.

Content theme	Feedback and changes	Comments by caregivers
Understandability and learnability	The term “full-blown” when used to refer to anxiety and depressive disorders was removed. The title Background Information was changed to Resources in the template.	“There were no unfamiliar terms to me. It was easy to read and comprehend and left me with no questions.” [AB]
Completeness	The main body of both health letters was made more concise, and more information was added about how to access resources while accounting for constraints that caregivers face (eg, time constraints and financial). The main body was shortened so that it fit entirely on the first page of each letter.	“I would like to know more about resources, such as group therapy that might be at a better rate, or maybe something online that is free?” [SL]
Relevance	Participants all found the content of both the anxiety and depression health letters very relevant. SL liked the focus on self-care in the depression letter. She found the assessment statements in both letters very relevant as well.	“I liked that it was relevant to the broader caregiving community.” [RM]
Usefulness	“Recognize your boundaries” was added to the SMART^a^ Tips section in the Depression health letter. Information about making social media connections with other caregivers was added to the main body, as suggested by RM.	“I found the letter very useful because caregiver anxiety is so common and so overlooked; it is nice to have some resources.” [SL]
Quality and credibility	The average response was *very satisfied* when caregivers were asked about the overall quality and reliability of the health letters. The references increased the quality and reliability of the letters [AB].	“I found the background information increased the credibility of the facts. I liked that the links were relevant to papers published in recent years.” [AB]

^a^SMART: specific, measurable, attainable, realistic, and time bound.

**Table 3:** Summary of the usability themes of the Keeping Hope and Stigma health letters’ content and feedback and changes made.

Content theme	Feedback and changes	Comments by caregivers
Understandability and learnability	The definition of stigma was clarified in the Stigma letter. The terms in the bullet list on page 2 of the Keeping Hope letter were bolded to stand out more and be more learnable and memorable.	“I learned that I need to educate others and get my story out there more to reduce the stigma.” [KL]
Completeness	Overall, participants liked the length and completeness of the letters. JM found the Stigma letter too negative. The letters were all edited to increase the use of positive, optimistic, and empowering statements, particularly in the SMART^a^ Tips sections. WM, CM, and JM wanted the point about connecting with a higher power, in the main body of the Keeping Hope letter, to be removed. This feedback was implemented.	“I found the letters provided a nice quick snippet of a little bit of information; even though they were short, there were some nice messages to take away.” [KL]
Relevance	JM felt that the letters should be made more targeted to specific types of caregivers so that they can be more relevant to peoples’ needs such as her own, given her son’s condition is quite rare. In the SMART Tips section of the Keeping Hope letter, CM did not find the “Make a positives and negatives list” and the “Set short-term goals” points to be relevant to caregivers of individuals with acquired brain injury because she felt it would be easier to just cope with things as they come along instead of risking overthinking about the future.	“It was very relevant because it touches on areas of hope that would have been especially helpful when I was in the most burdensome part of my caregiving experience.” [DM]; “The content should be made more relevant and targeted. How can you help someone who is in a completely different situation than somebody else?” [JM]
Usefulness	Additional links were added to the end of the health letters under the Resources section of the Keeping Hope and Stigma letters to make them more useful for caregivers needing more information on a specific topic.	“It was useful overall, but I would like to see contacts for if a person needed help with something.” [WM] (referring to the Keeping Hope letter)
Quality and credibility	The average response was *very satisfied* when caregivers were asked about the overall quality and reliability of the health letters.	—^b^

^a^SMART: specific, measurable, attainable, realistic, and time bound.

^b^Not applicable.

## Results

### Content

All caregivers made positive comments about the health letter content (see Tables 2 and 3), including the main body (first page), the image, the specific, measurable, attainable, realistic, and time-bound (SMART) Tips, the assessment, the Personal Account, and the Resources. Some minor, content-specific suggestions regarding each section were made. For example, RM felt that making major life changes during a stressful and tumultuous caregiving period would not be realistic for most caregivers. She felt that the actionable suggestion in the Anxiety letter “Limit caffeine and alcohol” would be a poor suggestion. However, she also acknowledged that this would depend on the severity and nature of the illness of the care recipient.

JM and DM both did not like the first suggestion in the SMART Tips section of the Keeping Hope letter, “Accept your situation and your role in it.” They found it too abrasive and confrontational.

In the Keeping Hope letter, JM and DM also did not like the last point in the SMART Tips section, “Identify and use your supports,” and the last bullet point in the first page (main body) section “Connecting with your outside support system...engage in community activities.” JM felt that these were not attainable or realistic because they are outside of the caregiver’s control: “You can try to connect with them, but if they aren’t there for you, they’re not, and you should go find support in other places, where people can relate, and they’re not scared of your situation.” JM said “you can do that if you have time, and if you live in a place where there are community activities, but a lot of people don’t. You need the time, the energy, the resources.”

JM also did not like the suggestion of the Keeping Hope letter: “Accept the things you cannot change, such as the course of your loved one’s illness.” JM found it too simplistic, negative, and “bossy” because “there’s some things you can change, but you don’t know it until you have enough information. This is why it is critical to persist to inform yourself as much as possible. That means finding people who can help you.” JM, WM, and CM did not like the suggestion of the Keeping Hope letter about connecting with a higher power because they felt that this would not be useful or relevant for many caregivers (Table 3).

AB, RM, and SK suggested modifying the font and colors of the figure in the Depression health letter and simplifying the figure’s text to improve its readability. ME suggested changing the title of the Background Information section to Resources to increase its understandability.

SL suggested the signs and symptoms of anxiety be placed on the first page of the Anxiety health letter. DM suggested that caregiver stigma be defined more clearly in the Stigma health letter.

Overall, caregivers were very pleased with the completeness of the health letters. However, a common theme regarding the completeness of the letters was that caregivers wanted more information on solutions, such as more coping skills, treatments, and resources for anxiety and depression (SL, RM, AB, and ME). Participants felt that the health letters should overall be more actionable. For example, RM said “Don’t tell people what they already know. Identify the issue and provide a solution!”

Furthermore, AB and SK suggested that both the Anxiety and the Depression letters deemphasize the role of antidepressant medications as a treatment. They did not want the health letter to make it seem like the first treatment option for anxiety or depression in caregivers is medication. In the list of treatment options, instead of antidepressants being listed first, talk therapies, *then,* caregiver support groups, and *finally,* antidepressant medications were listed.

SL suggested adding in more details to the Personal Account section about the name and age of each caregiver.

Participants found the health letter content quite useful. Although some caregivers did not find certain content relevant to their situations, they recognized that it might be relevant to caregivers more generally. For example, RM found the Anxiety letter content not relevant to her personally but stated that it would be relevant within the “broader caregiving community.” In addition, in the SMART Tips section of the Depression letter, RM suggested adding a tip about boundaries.

To make the content of the assessment more relevant in the Anxiety letter, several participants suggested including more response options in the scale. Instead of having just 4 response options, participants suggested adding an option between *not at all* and *several days*. An in-between rating was also suggested for the “Rate our Newsletter” section, between the *yes* and *no* options (Textbox 2).

Of all of the caregivers, JM and CM were the least satisfied (they selected the *moderately satisfied* option) with the usefulness of the health letters they read (the Keeping Hope and Stigma letters) because they did not think we could make a health letter that is relevant and useful for all types of caregivers, given that caregiver experiences are so diverse: “Trying to produce newsletters for a one-size-fits-all is going to be tough” (CM). It is also relevant to note that JM and CM were the most burdened caregivers in the sample based on the length of time they had spent caregiving in years (see Table 1).

The average response for the quality and credibility of the health letter content was *very satisfied* (see Tables 2 and 3). The Resources section helped to increase the credibility of the health letter content. Participants appreciated seeing the additional references. Even if they indicated they would not actually use them, they found it important that they be included. KL reported that she was extremely satisfied with the letter quality. She found the health letters better than any of the other materials she had read before.

**Changes made to the design and format (ie, to the template) of the series.**

- The font of the letters was changed to Arial (from Hoefler text; based on the suggestion by KL).
- The title, subtitle, and section headings were changed from blue and black to dark red (to increase consistency of the colors throughout the letter).
- The font of the titles was reduced to size 40 and bolded. The subtitle fonts were increased to size 18 and bolded (to reduce the size discrepancy between the title and subtitle and simplify the layout; based on the suggestions by CM, KL, AB, and WM).
- The Call to Action title was changed to SMART (specific, measurable, attainable, realistic, and time bound) Tips, bolded, and placed in all capital letters. Its font was increased to size 16.
- Each suggestion in the SMART Tips sections was bolded and increased to size 14. The black text explaining each suggestion was changed to font size 11 (not bolded).
- The entire letter template was changed to single spacing.
- The main body of text was made more concise, so that it all fit onto the first page.
- The title of the Background Information section was changed to Resources.
- The Rate Our Newsletter section title was changed to Rate Our Health Letter.
- Not applicable (N/A) was added as an option between yes and no in the Rate Our Health Letter section.
- The yes, N/A, and no response options in the Rate Our Health Letter section were adjusted so that they lined up for each of the 3 questions in this section.
- The first name and age of the caregiver were added in to each Personal Account (based on the suggestion by SL).

### Design and Format

Participants generally provided positive feedback about the design and the layout. Participants were satisfied with the length of the letters; 3 pages was the optimal length suggested by the majority of participants.

CM and KL suggested the design and layout of the health letters be simplified (Textbox 2). CM suggested the fonts be more consistent on the first page. This was in reference to the size discrepancy between the large red “90SECOND CAREGIVER” title and the small blue subtitle beneath it. AB, WM, CM, and DM suggested that the “90SECOND CAREGIVER” title be bolded, that the blue subheading beneath the title be a larger font, and that the SMART Tips section (on the first page) be more actionable. KL suggested that the font of the health letters be increased and changed to a more readable font, such as Arial (Textbox 2).

CM and WM felt that the SMART Tips should be on the left-hand side of the page at the beginning and that the main body should be underneath. On the contrary, SK suggested that the SMART Tips section be moved more to the right to make it clearer to the reader that the main body section should be read first.

RM, ME, and WM found that the SMART Tips provided a good summary of the content of each health letter, which was helpful because the reader could read this first to decide if they want to read the entire letter (they may not want to read it all if they do not find it relevant for their needs). ME also felt that having more of the main body section content in point form would make the material more readable, increasing its learnability.

All changes to the template of the series (as opposed to minor, content-specific changes) were implemented only upon consultation and agreement among the principal investigator, the editor in chief, and the principal scientist of the series (Textbox 2).

### Likes and Dislikes (Secondary Objective)

Regarding the format and content, participants did not like that only 4 response options were present in the rating scale for the Anxiety health letter. Overall, participants liked that each main body section was comprehensive, providing a good overview of each topic and referring to real-world statistics. However, some participants mentioned they would like the main body section to be more oriented toward solutions to anxiety and depression rather than explaining the problem itself. Participants also enjoyed reading the Personal Account section. RM stated, “it is always interesting to hear other peoples’ stories.” Participants appreciated the fact that references were included because this increased the overall quality and credibility of the health letters.

RM did not like the suggestions in the Depression letter (relating to sleep, social activities, and self-care) because she thought they would be too unrealistic and unattainable for caregivers in situations such as her own, caring for an acutely and severely ill child with extended, frequent hospital stays. However, RM was one of the most burdened caregivers in the sample (60 hours of caregiving per week), so her situation may not be generalizable to most caregivers.

Overall, the SMART Tips and the Personal Account were the sections of the letters that participants liked the most. KL stated that the Personal Account “validates how people are feeling and what they are dealing with.”

Regarding the main body section of the Keeping Hope letter, JM said: “it is burdensome for caregivers to be reading such negative content. There are too many negative elements that aren’t helpful.” She also did not like the assessments, because she did not feel they could add anything in terms of addressing caregiver burden and the burden of stigma (particularly in the Stigma health letter). However, JM enjoyed the Personal Account section and the SMART Tips, particularly the location of the SMART Tips within the health letters, and its clarity. She also had quite a few specific suggestions to help improve each SMART Tips point in both health letters that she read. For example, she felt that the first point in the SMART Tips section of the Stigma letter “Educate others” might not be specific enough or attainable because she believes that most people are not open and willing to listen, and this advice would be more difficult for introverted caregivers to follow. She also felt the second point in the SMART Tips of the Stigma letter, “Don’t be Shy...Make sure to bring your loved one to gatherings” was too simplistic and unrealistic (ie, there are too many barriers to caregivers actually implementing this advice). She felt that telling a caregiver who is introverted “don’t be shy” is like telling someone with depression to just “get over it.”

JM and DM both did not like the graphic in the Keeping Hope letter because they felt it was too dark and pessimistic.

### Additional Topics Suggested (Secondary Objective)

Textbox 3 lists all the additional topics that participants mentioned that they would like to see if they were subscribers to the series in the future. Furthermore, all the caregivers, except for 1 (CM), expressed an interest in becoming subscribers to the 90Second Caregiver series in the future.

**Additional topics suggested.**

- Caregivers of the sandwich generation
- Caregiving and finances
- Caregiving for children
- Perfectionism in caregivers
- Insomnia in caregivers
- Faith in caregivers
- Social isolation in caregivers
- Caregiver support groups or the importance of a support system in caregivers
- Caregiver burnout
- How to actively participate in your loved one’s health care
- How to balance caregiving with being a parent
- How to deal with setbacks or relapse in recovery
- How to empower and educate yourself as a caregiver
- How to engage your loved one (the care recipient) in their health care
- Finding resources for help as a caregiver
- How to handle negativity as a caregiver, especially in the care recipient
- Supporting an adult survivor of acquired brain injury, for example, with independence, romantic relationships, and workplace discrimination

## Discussion

### Principal Findings

The main outcome of the study was to assess and consequently improve the usability of 90Second Caregiver based on caregiver input. Usability in this study was assessed in terms of the themes of understandability, learnability, completeness, practical relevance, usefulness, and quality and credibility of the health letter content, as well as design and format. Findings reinforced the fact that 90Second Caregiver is a very user-friendly, learnable, and useful series of health letters. Caregivers were very satisfied with both the content and design of the series. Participants found the reading level acceptable (eg, no unfamiliar terms), and they found the content provided an excellent summary of each health letter topic. The majority of participants found the information useful and relevant to their needs as caregivers, and they were satisfied with the content’s credibility.

Some letter-specific changes as well as template changes were suggested and implemented based on the feedback data of participants. On the basis of participant feedback, the content of the health letters will be changed to specify that the letters should (1) contain specific, measurable, attainable, realistic, and time-bound tips; (2) be objective, empowering, positive, and optimistic; (3) avoid overly *bossy* language or polarizing topics; (4) avoid suggesting changes that require significant financial investment or travel time; and (5) ensure that the main body section is solution oriented instead of explanation based. Textbox 2 provides a summary of the changes implemented to the template of the series based on participant feedback and the principles of persuasive design, such as making sure that the SMART tips are fitting suggestions that can be used successfully by caregivers and that the health letters are visually attractive in addition to being trustworthy [20].

A relationship was observed between the perceived usefulness of the letters and the burden of caregiving in this sample. Caregivers who were more burdened (RM, JM, and CM), based on the length of time they spent caregiving per week and/or the length of time in years that they had been caregiving, tended to be more critical of the health letter content, finding it less useful and relevant. For example, RM was one of the most burdened caregivers of the sample, spending 60 hours a week caregiving, so her situation may not necessarily be the most representative of a typical caregiver or subscriber to 90Second Caregiver.

If this finding is confirmed with a larger sample, it may be best to target the current letters toward individuals who are relatively new to caregiving or part-time caregivers. In addition, a different approach may be needed for caregivers who are more burdened because the caregivers who were overly critical tended to find the content of the letters less useful and feasible. This new approach could be needed because these caregivers may simply be too burnt out from the demands of caregiving to truly appreciate, retain, and apply the health knowledge in the letters, or they may be so experienced in their caregiving role that they find the letters redundant. To better address the needs of these caregivers, a new approach could involve pairing the 90Second Caregiver letters with another distance-delivered intervention, such a Web-based weekly stress-management health letter.

Many participants commented that they appreciated being included in this project and provided with the opportunity to vocalize their feedback and sharing how it was related to their specific caregiving experiences.

### Limitations and Strengths

One limitation of this study is that social desirability bias might have influenced the way that participants responded to the interview questions. In other words, participants may have overemphasized the positive features of the health letters and omitted certain usability issues or factors that they disliked about the letters to please and impress the interviewer [21]. This potential bias was minimized by verbalizing to participants to not worry about *hurting the investigator’s feelings* and encouraging them to give their honest thoughts during the interview. Confirmation bias, the tendency to interpret data in a way that confirms the researchers’ pre-existing beliefs about the usability of the health letters, might have also been a risk to the validity of the results [21]. This potential bias was addressed by having a second researcher review the usability themes and categories that emerged from the thematic analysis of the interview transcripts.

Another limitation was that not all the changes proposed by the caregivers were able to be implemented because of inconsistencies in the feedback provided [13]. A larger sample size may have clarified some of these inconsistencies, but it was not possible to implement it in this study because of time constraints.

Another limitation was that more than half of the participants had a university qualification and/or worked in a health-related field. This could explain why some of the participants were overly critical of both the design and the content of the letters.

A significant strength of this study was that triangulation (ie, both the concurrent think aloud method and retrospective questioning) were used to assess caregivers’ usability experiences [16,22]. Triangulation is a means to assess outcomes from multiple perspectives to ensure that the usability findings are reliable (ie, consistent, reproducible, and repeatable) and valid (trustworthy, credible, and accurate). Another strength was that caregivers in our sample represented a broad range of ethnic and sociodemographic groups, with a variety of caregiving backgrounds and experiences.

### Conclusions

In conclusion, the findings showed that health information for caregivers is most usable when it is delivered in a solution-oriented (as opposed to a fact-based) manner. Incorporating principles of SMART goals may also be useful to improve usability.

The results of this study may be the building blocks for the examination of other eHealth information formats, making them valuable to future researchers testing the usability of health information products. The approach that we took in designing the 90Second Caregiver letters, and in subsequently testing their usability, has 3 main benefits. First, it appears to be useful for examining the format and delivery of health information (which is an understudied domain in health research). Second, it is useful for usability hypothesis generation (however, our present 90Second Caregiver design and delivery approach may not be useful for highly burdened caregivers or those who have been caregiving for many years). Third, our approach can generate information that is useful for a similar set of products (ie, health letters targeted toward other conditions/populations, such as 90Second Parent or 90Second Cannabis), even though we used a relatively small sample from our larger repertoire of health letters for this study.

## Abbreviations

eHealth: electronic health
SMART: specific, measurable, attainable, realistic, and time bound
